# Commentary: Newly diagnosed hepatic encephalopathy presenting as non-convulsive status epilepticus: a case report and literature review

**DOI:** 10.3389/fneur.2025.1731471

**Published:** 2025-12-15

**Authors:** Philippe Gélisse, Arielle Crespel

**Affiliations:** 1 Epilepsy Unit, Hôpital Gui de Chauliac, Montpellier, France; 2 Research Unit (URCMA: Unité de Recherche sur les Comportements et Mouvements Anormaux), INSERM, U661, Montpellier, France

**Keywords:** nonconvulsive status epilepticus, hepatic encephalopathy, electroencephalogram (EEG), neuroimaging, Salzburg criteria, benzodiazepines (BZDS)

**Figure 1:**
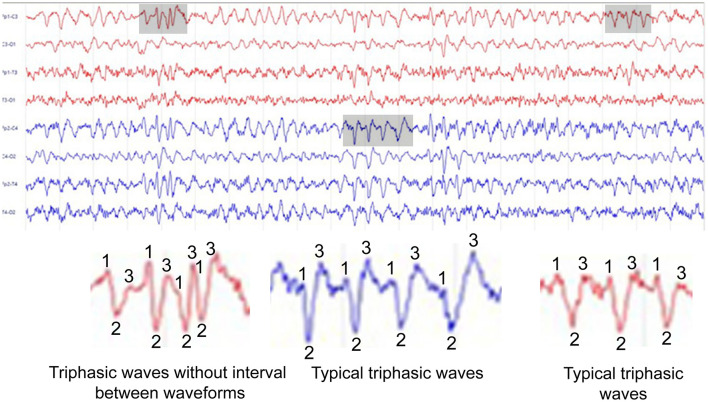
This EEG, adapted from Olivero et al., was originally labeled as status epilepticus. However, it demonstrates bilateral runs of triphasic waves (TWs), a pattern characteristic of metabolic encephalopathy—specifically hepatic encephalopathy in this case. The shaded (gray) areas include zoomed-in segments to highlight the three distinct phases of the waves, which are annotated for clarity. In the first panel, the TWs appear continuously, with no discernible intervals between successive waveforms.

Olivero et al. described a case of hepatic encephalopathy (HE) presenting as nonconvulsive status epilepticus (NCSE), asserting that this was the first reported instance of NCSE as the initial manifestation of HE (1). It is a typical HE case without any ictal activity on the EEG. Indeed, their diagnosis of NCSE based on EEG findings is very questionable. The authors interpreted the EEG as demonstrating “*fast paroxysmal bilateral sharp-wave activity*” that resolved after diazepam administration. Yet, the provided EEG recordings are more consistent with metabolic encephalopathy, characterized by runs of triphasic waves (TWs) (Figure 1) associated with slow-wave activity. This strictly corresponds to the HE pattern described by Bickford and Butt 70 years ago (2).

The observed EEG improvement following diazepam does not confirm NCSE, as diazepam is a hypnotic agent. There is a common false syllogism in the case of TWs: “Intravenous benzodiazepines *suppress the ictal activity in NCSE, TWs are suppressed by intravenous benzodiazepines. Therefore, these patients have NCSE*” (3). In the case report of Olivero et al., the resolution of TWs simply reflects sleep induction rather than seizure termination. Older studies have shown that TWs and rhythmic delta waves in metabolic encephalopathy either decrease or disappear entirely during NREM sleep (4). In his famous Textbook of Electroencephalography, Niedermeyer, wrote of HE that “*when such patients are allowed to fall asleep, normalization of the record takes place for the duration of sleep*” (5). In this chapter, two examples of TW runs closely resemble those of Olivero et al.

Authors should incorporate EEG reactivity testing into their clinical protocols for patients with suspected NCSE, as it offers critical diagnostic insights beyond conventional criteria. This simple, cost-effective test is a safer alternative to empirical trials of antiseizure medications. In true NCSE, epileptiform activity is self-sustaining and typically unresponsive to external stimuli. In contrast, TWs or generalized periodic discharges in metabolic/toxic encephalopathies often exhibit vigilance-dependent reactivity. This resolves transiently when patients are aroused from drowsiness to full wakefulness, although reactivity tends to disappear with the increasing severity of the disease and in comatose patients. In patients without preexisting epileptic encephalopathy, stimulus-induced wakefulness with transient EEG improvement strongly favors a non-ictal (encephalopathic) pattern over NCSE (Table 1) (6).

**Table 1:** Nonconvulsive status epilepticus vs. metabolic/toxic encephalopathies with generalized periodic activity.

**Question 1**	**Is it a confusional state or only a problem of vigilance?**
Question 2	Is there a fluctuation of symptoms or a change in consciousness from somnolence to coma?
Question 3	Is the EEG activity rhythmic or periodic?
Question 4	Is the EEG activity dynamic, showing spatiotemporal evolution, or relatively monomorphic?
Question 5	Is the EEG reactive to stimuli, wakefulness, sleep, arousal, or antiseizure drugs^*^?
Question 6	Neuroimaging results

The six key questions. ^*^Only consider an IV benzodiazepine test positive if both EEG and consciousness normalize.

Moreover, the authors reported unremarkable CT perfusion findings, whereas MRI revealed changes consistent with hepatic encephalopathy but no evidence of status epilepticus (SE). However, they based their diagnosis of SE solely on EEG results, disregarding the potential diagnostic value of neuroimaging. Recent studies increasingly highlight the utility of CT perfusion and MRI —particularly with arterial spin labeling sequences—in detecting NCSE (7–11), especially in focal SE. The authors did not specify whether their case involved focal or generalized SE, noting only a right-hemispheric predominance of abnormalities in the fronto-temporal region—a finding not clearly supported by their EEG. The paroxysmal activity (TWs) depicted in their figure appears bilateral, raising questions about the lateralization described.

Patients with uremic or other toxic encephalopathies have seizures more frequently than those with hyperammonemic encephalopathy, reflecting diffuse cortical hyperexcitability. Nevertheless, seizures can occur in HE as well. We report a 66-year-old male with alcohol-induced cirrhosis who was hospitalized in coma. His EEG demonstrated independent right- and left-hemispheric focal subclinical seizures, and a CT scan showed cerebral edema. Because the seizures were not recognized, his course progressed to a pattern consistent with anoxic encephalopathy (12).

In conclusion, sometimes the EEGs of patients with metabolic/toxic encephalopathy are striking, and NCSE may be part of the differential diagnosis. In addition to the Salzburg criteria for diagnosing NCSE, six key questions should be routinely considered in such cases (Table 1). Now, neuroimaging findings have become an essential component in refining the diagnostics of NCSE.
